# Secretome Analysis of Human and Rat Pancreatic Islets Co-Cultured with Adipose-Derived Stromal Cells Reveals a Signature with Enhanced Regenerative Capacities

**DOI:** 10.3390/cells14040302

**Published:** 2025-02-18

**Authors:** Erika Pinheiro-Machado, Bart J. de Haan, Marten A. Engelse, Alexandra M. Smink

**Affiliations:** 1 Department of Pathology and Medical Biology, University Medical Center Groningen, University of Groningen, Hanzeplein 1, 9713 GZ Groningen, The Netherlands; 2 Leiden Transplant Center, Leiden University Medical Center, Albinusdreef 2, 2333 ZA Leiden, The Netherlands

**Keywords:** pancreatic islets, adipose-derived stromal cells, co-culture, proteomics

## Abstract

Pancreatic islet transplantation (PIT) is a promising treatment for type 1 diabetes (T1D) but faces challenges pre- and post-transplantation. Co-transplantation with mesenchymal stromal cells (MSCs), known for their regenerative properties, has shown potential in improving PIT outcomes. This study examined the secretome of islets cultured alone compared to the secretomes of islets co-cultured with adipose-derived stromal cells (ASCs), a subtype of MSCs, under transplantation-relevant stressors: normoxia, cytokines, high glucose, hypoxia, and combined hypoxia and high glucose. Islet co-culture with ASCs significantly altered the proteome, affecting pathways related to energy metabolism, angiogenesis, extracellular matrix organization, and immune modulation. Key signaling molecules (e.g., VEGF, PDGF, bFGF, Collagen I alpha 1, IL-1α, and IL-10) were differentially regulated depending on culture conditions and ASC presence. Functional assays demonstrated that the co-culture secretome could enhance angiogenesis, collagen deposition, and immune modulation, depending on the stress conditions. These findings highlight possible mechanisms through which ASCs may support islet survival and function, offering insights into overcoming PIT challenges. Moreover, this work contributes to identifying biomarkers of the post-transplantation microenvironment, advancing therapeutic strategies for T1D and regenerative medicine.

## 1. Introduction

Pancreatic islet transplantation (PIT) remains a promising treatment for type 1 diabetes (T1D), though its clinical success is frequently hindered by both pre- [1,2] and post-transplantation challenges [3,4]. Long-term efficacy is often compromised by limited engraftment due to insufficient vascularization, extracellular matrix (ECM) degradation, and immune-mediated rejection [3,4,5]. Addressing these challenges is essential for extending the benefits of PIT to a broader patient population. To overcome the loss of islet quality and poor survival rates, researchers have explored co-culturing or co-transplanting islets with mesenchymal stromal cells (MSCs) [6,7,8,9]. MSCs are a subset of heterogeneous non-hematopoietic fibroblast-like cells with multi-lineage differentiation capacity [10,11]. These multipotent MSCs can be obtained from various sources, such as adipose tissue (adipose-derived stromal cells (ASCs)), contributing to tissue repair through migration and secretion of biologically active molecules with anti-inflammatory, immunomodulatory, and angiogenic properties [12,13,14,15]. Several studies, both in vitro and in T1DM animal models, have demonstrated the effectiveness of co-culturing/co-transplantation of islets with MSCs, including ASCs, showing enhanced insulin secretion, reduced beta cell apoptosis, and increased beta cell mass [6,7,9,16,17,18,19,20]. However, the mechanisms behind the positive PIT effects of MSC, particularly ASC, co-transplantation with islets remain unclear. It is hypothesized that these improvements could result from either direct contact between islets and MSCs or the production of biologically active molecules (the secretome).

To bridge this knowledge gap, the primary goal of this study was to explore the secretome profile of human and rat islets cultured alone versus those co-cultured with ASCs under multiple conditions that replicate the transplantation environment: normoxia, hypoxia, high glucose, cytokine exposure, and hypoxia combined with high glucose. By investigating whether ASC co-culture influences pathways critical to transplantation success—angiogenesis, ECM organization, and immune modulation—we aimed to uncover potential mechanisms that promote islet survival and functional stability in the post-transplant setting. The inclusion of both human and rat models allowed us to investigate potential species-specific differences while focusing on the broader utility of secretome analysis to assess the functional impact of ASCs on islet health. While rat models are commonly used in preclinical studies, human data are critical for direct application in therapeutic contexts. By including both species, we bridged the gap between basic research and clinical translation, ensuring that the findings from animal models are relevant for human therapies, particularly in the fields of PIT and regenerative medicine.

Furthermore, our chosen method was designed to capture a broad overview of the secretome and identify patterns and trends in protein expression across groups. By analyzing the complete list of proteins, we aimed to provide a comprehensive view of the biological processes potentially involved, which we believe is essential during this exploratory phase. Moreover, the data generated in this study constitute a database that can serve as a valuable resource for identifying biomarkers of the microenvironment post-(co-)transplantation and offers a basis for further exploration of key pathways and targets that may optimize ASC–islet co-transplantation strategies. Overall, our findings provide insights into the potential of ASCs to improve transplant outcomes and advance the clinical application of PIT.

## 2. Materials and Methods

### 2.1. Experimental Design

**Figure 1:**
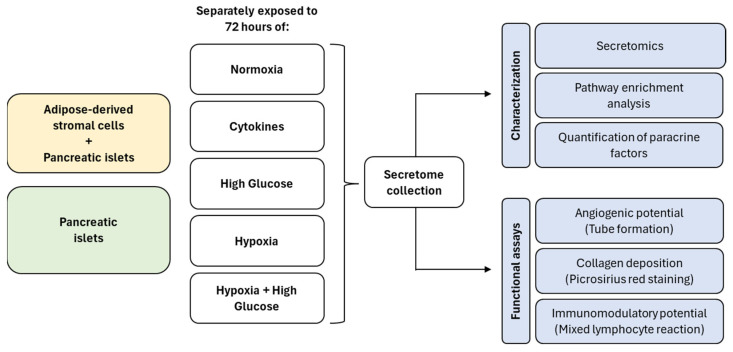
A flow diagram of the experimental design.

In this study, the changes in the secretion profiles of human and rat pancreatic islets cultured alone or in co-culture with human or rat perirenal ASCs (h-prASCs or r-prASCs) were explored when subjected to various conditions designed to induce a different type of stress, mimicking challenges encountered during isolation and PIT. These conditions included normoxia (as the baseline control), exposure to cytokines, high glucose, hypoxia, and a combination of hypoxia and high glucose (Figure 1). Co-culturing was investigated in two distinct ratios of islets to ASCs, 1:300 and 1:1000. Islets and co-cultures were subjected to these conditions for 72 h (h). Ultimately, the supernatants (secretomes) were collected and analyzed using mass spectrometry, Luminex, and an enzyme-linked immunosorbent assay (ELISA). The secretion profiles were characterized, and pathway enrichment was described. To validate the in silico findings, we conducted in vitro functional investigations, focusing on the secretome’s capacity to stimulate critical processes, such as angiogenesis, ECM deposition, and the attenuation of alloimmunity. These were achieved by performing a tube formation assay (TFA), Picrosirius Red staining, and a one-way mixed lymphocyte reaction (MLR) followed by an antibody-mediated cell-dependent cytotoxicity assay (CDC).

### 2.2. Human and Rat Perirenal Adipose Tissue

This study used human and rat donors to obtain ASCs, as previously described by Pinheiro-Machado, E. et al. [21]. Human perirenal adipose tissue was procured from living kidney donors (n = 5) at the University Medical Center Groningen (UMCG; Groningen, The Netherlands). In the Netherlands, it is legally permitted to use residual materials from patient care for scientific research without explicit consent, provided the materials are collected anonymously (as was the case for the human adipose tissue) and the patient has not raised any objections (which was always verified). Donors comprised males (43%) and females (57%). The average age was 57.6 ± 11.5 years. Maintained at 4 °C, the human samples were processed within a 48 h time window. In turn, rat perirenal adipose tissue was collected from 7–9-week-old male Sprague-Dawley rats (n = 4; Envigo, Horst, The Netherlands). Ethical approval for tissue collection and procedures was obtained from the Dutch Central Committee on Animal Testing and the University of Groningen’s Animal Welfare Authority (AVD10500202115138).

### 2.3. Medium Preparation for Culturing and Secretome Collection

For the prASC isolation, a standard medium without serum (STD-M (−)) was used. The STD-M (−) consisted of Dulbecco’s Modified Eagles Medium 4.5 g/L D-glucose (DMEM; Lonza, Walkersville, MD, USA) supplemented with 50 U/mL penicillin (Thermo Fisher Scientific, Bleiswijk, The Netherlands), 50 µg/mL streptomycin (Thermo Fisher Scientific), and 2 mM L-glutamine (Thermo Fisher Scientific). For the prASC and fibroblast culturing, a standard medium containing serum (STD-M (+)) was used. STD-M (+) contains DMEM 4.5 g/L D-glucose supplemented with 50 U/mL penicillin, 50 µg/mL streptomycin, 2 mM L-glutamine, and 10% heat-inactivated fetal bovine serum (FBS; Thermo Fisher Scientific). For culturing the islets, Connaught Medical Research Laboratories (CMRL) medium containing serum (CMRL-M (+)) was used. The CMRL-M (+) consisted of CMRL medium (Thermo Fisher Scientific) containing 8.3 mM glucose and supplemented with 10% FBS, 2 mM GlutaMax supplement (Thermo Fisher Scientific), 50 U/mL penicillin, and 50 µg/mL streptomycin. For harvesting the normoxia- and hypoxia-derived secretomes from the islets and co-cultures, CMRL without serum CMRL-M (−) was used. CMRL-M (−) consisted of CMRL medium (8.3 mM glucose) supplemented with 2 mM GlutaMax supplement, 50 U/mL penicillin, and 50 µg/mL streptomycin. The cytokine-derived secretomes were collected in CMRL-M (−) supplemented with 50 ng/mL interferon-gamma (IFN-γ) (ImmunoTools, Friesoythe, Germany), 21.5 ng/mL tumor necrosis factor-alpha (TNF-α) (ImmunoTools), and 0.25 ng/mL interleukin 1 beta (IL-1β) (ImmunoTools). Human and rat cytokines were obtained from ImmunoTools, and the same concentrations were used for both species. The high-glucose- and hypoxia + high glucose-derived secretomes were collected in CMRL-M (−) adjusted to 16.7 mM glucose. RPMI (+) (RPMI 1640 (Thermo Fisher Scientific)) containing 4.5 g/L D-glucose, supplemented with 50 U/mL penicillin, 50 µg/mL streptomycin, 2 mM L-glutamine, and 10% FBS, was used to culture human peripheral blood mononuclear cells (PBMCs) and rat splenocytes. For the MLR, RPMI (−) (RPMI 1640 without FBS) was used as a control.

### 2.4. prASC Isolation and Culture

Both rat and human prASCs were isolated following the protocol previously described [21]. Briefly, the tissue was washed, minced, and digested in a standard medium depleted of serum (STD-M (−)) containing NB 4 collagenase (0.5 mg/mL; Nordmark Biochemical, Uetersen, Germany) for 30 min (min) at 37 °C. Cells were then separated from debris via centrifugation (500× *g*, 5 min), and the resulting pellet was resuspended in STD-M (+). Resuspended cells were plated in T25 flasks for initial cell culture [passage 0] at 37 °C and 5% CO_2_. After 24 h, debris and non-adherent cells were removed, and fresh STD-M (+) was added to the adherent cells. This medium was replaced every three days. At 80% confluence, cells were passaged by trypsinization. They were then expanded until passage 3 (h-prASCs) or 2 (r-prASCs) and stored in liquid nitrogen until they were needed for the co-culture experiments with rat and human islets. Both human and rat prASCs have been previously characterized [21].

### 2.5. Human Pancreatic Islets

Human islets were procured either from Leiden University Medical Center (LUMC; Leiden, The Netherlands) or the European Consortium for Islet Transplantation (ECIT; Milan, Italy) [22,23]. Detailed information about the donors’ and islets’ characteristics can be found in Table 1. Research consents were obtained following the respective centers’ national regulations. The islets, which did not meet the strict quality and/or quantity criteria necessary for clinical use, were allocated for research use only. Upon arrival at the UMCG, the islets were hand-picked to maximize purity. Selected islets were then cultured in CMRL-M (+) for 24 h in an incubator set at 37 °C and 5% CO_2_ to allow for acclimatization. After this period, the islets were cultured further for secretome collection.

### 2.6. Rat Pancreatic Islet Isolation

Rat islets were isolated from the same animals that served as donors for the r-prASCs (described above). The isolation procedure followed a previously described protocol [24]. Briefly, the rat pancreas was distended by injecting ± 9 mL collagenase V solution (1 mg/mL; Sigma-Aldrich, Zwijndrecht, The Netherlands) in Hank’s Balanced Salt Solution (HBSS; Gibco, Thermo Fisher Scientific) via the pancreatic duct. Subsequently, the isolated islets were washed and purified using a Ficoll density gradient (Gradient stock solution; Corning Cellgro, Manassas, VA, USA). Purified islets were cultured overnight in CMRL-M (+) at 37 °C and 5% CO_2_. Acclimatized islets were used within 24 h for secretome collection.

### 2.7. Secretome Collection

For the co-culture conditions, h-prASCs or r-prASCs were seeded at 75,000 or 250,000 cells/well in non-adherent 6-well plates (Thermo Fisher Scientific) containing STD-M (+). These cell concentrations were determined using the ratios 1:300 and 1:1000 (islets to prASCs). Plated cells were incubated overnight in a 5% CO_2_ incubator at 37 °C to allow adherence. After this period, adherent cells were carefully washed with phosphate-buffered saline (PBS) (Thermo Fisher Scientific), and 250 islets were added to each well. For the collection of secretomes, the islets (250) or the co-cultures of islets and prASCs (1:300 and 1:1000) were cultured in CMRL (−)—as this medium is the standard medium used for islet culturing—under the following conditions: normoxia (21% O_2_, 5% CO_2_), cytokine exposure (21% O_2_, 5% CO_2_, 50 ng/mL IFN-γ, 21.5 ng/mL TNF-α, 0.25 ng/mL IL-1β), high glucose exposure (21% O_2_, 5% CO_2_, 16.7 mM glucose), hypoxia (1% O_2_, 5% CO_2_), and hypoxia + high glucose exposure (1% O_2_, 5% CO_2_, 16.7 mM glucose). After 72 h, the secretomes were harvested, pooled into three pools of three donors, and subjected to centrifugation (500× *g*, 5 min) to remove undesired components. The 72 h culture duration and the 1:300 or 1:1000 co-culture ratios were selected based on previously published studies [6,7,19] indicating that these conditions optimize the interaction between islets and ASCs. Secretomes were then aliquoted and snap-frozen in liquid nitrogen before being stored at −80 °C until processing.

### 2.8. Discovery-Based Proteomics and Data Analysis

The protein composition of the various collected secretomes was determined using a discovery-based proteomics approach called label-free quantification [25]. Briefly, in-gel digestion was performed on 30 µL of the provided secretomes using trypsin (300 ng sequencing grade modified trypsin V5111; Promega, Leiden, The Netherlands) after first reducing them with 10 mmol/L dithiothreitol and then alkylating them with 55 mmol/L iodoacetamide proteins [26]. Discovery mass spectrometric analyses were executed on a quadrupole orbitrap mass spectrometer equipped with a nano-electrospray ion source (Orbitrap Exploris 480; Thermo Fisher Scientific). Peptides were chromatographically separated using liquid chromatography (LC) on an Evosep system (Evosep One; Evosep, Odense, Denmark) with a nano-LC column (EV1137 Performance column 15 cm × 150 µm, 1.5 µm, Evosep). The LC–mass spectrometry (LC-MS) procedure used the equivalent of 1 µL starting injection material, and the peptides underwent separation using the 30SPD workflow (Evosep). The mass spectrometer was operated in positive-ion mode and utilized the data-independent acquisition (DIA) mode with isolation windows of 16 *m*/*z*. The precursor mass range was set between 400 and 1000, and the FAIMS switched between CV-45 V and -60 V, with three scheduled MS1 scans during each screening of the precursor mass range. The LC-MS raw data underwent processing using Spectronaut (version 17.1.221229; Biognosys Inc., Cambridge, MA, USA), following the standard settings of the direct DIA workflow. Quantification was conducted on MS1, utilizing a human or rat SwissProt database (www.uniprot.org, containing 20,350 entries for human samples and 8094 entries for rat samples). Local normalization was applied for quantification, and Q-value filtering was set to the classic setting without imputation. The final list of proteins identified in all conditions can be found in Supplementary File S1, Table S1 (human), and Supplementary File S2, Table S1 (rat).

### 2.9. Mass Spectrometry In Silico Analysis

To gain insights into the potential functional changes that occur when islets and co-cultures are exposed to the various culturing strategies, the lists of proteins resulting from the secretomic studies were submitted to Metascape [27]. Metascape analyzed pathway enrichment clusters from Gene Ontology (GO) biological processes for each secretome. The top 100 biological processes from each secretome can be found in Supplementary File S1 (human), which contains 13 supplementary tables (i.e., ranging from Supplementary File S1, Table S1, to Supplementary File S1, Table S13), and Supplementary File S2 (rat), which contains 13 supplementary tables (i.e., ranging from Supplementary File S2, Table S1, to Supplementary File S2, Table S13).

### 2.10. Quantification of Paracrine Factors

Within the secretomes, paracrine factors linked to angiogenesis (vascular endothelial growth factor (VEGF), platelet-derived growth factor AB (PDGF), and basic fibroblast growth factor (bFGF)), as well as the ECM constituent (collagen I alpha 1) and immunomodulatory proteins (IL-10 and IL-1α) were measured using ELISA or Luminex techniques [21]. Levels of rat and human bFGF, PDGF, VEGF (DuoSet ELISA; R&D Systems, Abingdon, UK), and rat collagen I alpha 1 (Novus Biologicals, Biotechne, Minneapolis, USA) were quantified through ELISA. Rat and human IL-10, IL-1α, and human collagen I alpha 1 were assessed utilizing magnetic Luminex^®^ Assays (R&D systems; #LXSARM-03/#LXSAHM-04). The secretome samples used for these assays consisted of three samples from each culturing group that were pooled (n = 3). These were then measured in triplicate following the respective manufacturer’s protocols. For magnetic Luminex^®^ Assays, plates were analyzed utilizing a Luminex 200 System, with data analysis conducted through Luminex xPONENT software. For ELISA kits (R&D Systems and Novus Biologicals), data acquisition was performed using a microplate spectrophotometer (Epoch 2; BioTek, Winooski, VT, USA) at wavelengths of 450 nm, with 540 nm correction applied.

### 2.11. Tube Formation Assay

To assess the proangiogenic capacity of the human secretomes, the Endothelial Cell Facility of the UMCG provided commercially obtained human umbilical vein endothelial cells (HUVECs). The HUVECs were cultured in endothelial cell growth basal medium (EGM-2; Lonza) supplemented with EGM-2 MV SingleQuot Kit Supplements and Growth Factors (Lonza) at 37 °C with 5% CO_2_. Similarly, to assess the proangiogenic potential of rat secretomes, rat aortic endothelial cells (RAECs) were isolated according to a protocol previously outlined by Suh et al. [28]. For the assay, HUVECs (n = 3) and RAECs (n = 3) were seeded onto growth factor-reduced Matrigel (Corning, Amsterdam, The Netherlands) in a 96-well plate at a density of 35,000 cells per well. Secretomes were added, and cells were incubated for 18 h at 37 °C with 5% CO_2_. Post-incubation imaging was conducted using a Leica MZ7.5 microscope with a Leica IC90 E camera (Leica Microsystems B.V., Amsterdam, The Netherlands). HUVECs and RAECs cultivated in CMRL (−) served as controls. The angiogenic potential was quantified based on the number of branching points counted by Fiji software [29]. Subsequently, the number of branching points was normalized to the control condition (CMRL (−)), set at 1.

### 2.12. Picrosirius Red Staining

To assess the human secretomes’ capacity to induce collagen deposition, commercially acquired human fetal lung fibroblasts (FLF92, passage 28–30) were cultivated in STD-M (+). Comparatively, rat dermal fibroblasts (RDFs) were utilized to assess the potential of the rat secretomes to stimulate collagen deposition. RDF were obtained through a serial explant technique, as previously outlined by Nejaddehbashi et al. [30]. Picrosirius Red staining (Direct Red 80; Sigma-Aldrich) was performed to detect and quantify collagen deposition by FLF92 and RDF cells upon 72 h exposure to the different human or rat secretomes. The protocol of Xu et al. [31] was adapted for this purpose. The staining’s optical density (OD) was determined at 540 nm using a spectrophotometer. OD values were normalized to the total cell count (Countess 3 Cell Counter, Thermo Fisher Scientific) following exposure to each secretome. Collagen content is expressed as OD per 10,000 cells, with ODs standardized to the control (CMRL (−)), set at a reference value of 1.

### 2.13. One-Way MLR Followed by an Antibody-Mediated Cell-Dependent Cytotoxicity Assay (CDC)

To elucidate the immunomodulatory capacity of both human and rat secretomes, we performed an adaptation of a previously described one-way MLR followed by an antibody-mediated CDC method [32,33]. An overview of the procedure is depicted in Supplementary Figure S1. For human secretomes, human PBMCs were used. For rat secretomes, rat splenocytes were used.

#### 2.13.1. Human Peripheral Blood Mononuclear Cells (PBMCs)

To test the effects of different h-prASCs, isolated human PBMCs were used. To this end, buffy coats of healthy blood donors were obtained from the Sanquin Blood Bank (Groningen, The Netherlands). A Lymphoprep density gradient (Fresenius Kabin Norge AS, Oslo, Norway) and centrifugation (800× *g*, 15 min, room temperature (RT)) were used to obtain the PBMCs. Subsequent centrifugation to wash the cells was carried out at 300× *g* (5 min, RT). The supernatant was discarded, and the interphase consisting of PBMCs was transferred to new centrifuge tubes and washed three times with PBS. Before the final centrifugation, the PBMCs were filtered through a 100 µm cell strainer (FALCON, Corning, Durham, NC, USA). The viable cells were counted and immediately used for the MLR.

#### 2.13.2. Rat Splenocytes

To investigate the immunomodulatory potential of the different r-prASC secretomes, rat splenocytes were isolated from two distinct strains: Sprague-Dawley rats (the same animals used for adipose tissue collection, as previously described [34]) and Wistar rats. The samples from both strains were processed identically. Briefly, the spleens were cut into small pieces and mechanically disrupted in ice-cold RPMI (+) (Gibco). Splenic red blood cells were eliminated by incubation with ice-cold ammonium chloride (4 mL, 10 min; UMCG pharmacy). Falcon tubes with cell strainer caps (35 μm; Corning) were used to remove cell clumps before the cells were counted and immediately plated for the MLR.

#### 2.13.3. One-Way MLR Followed by CDC

The assay involved a one-way MLR, where stimulator and responder cells were co-cultured to generate alloantibodies. For the assay involving human cells, PBMCs from mismatched human donors were co-cultured. In the case of rat cells, the co-culturing was conducted using splenocytes obtained from two different rat strains, with Sprague-Dawley rat cells serving as stimulators/resting cells and Wistar rat cells serving as responders. Briefly, stimulator cells (0.5 × 10^6^ cells per well) were treated with mitomycin C (50 μg/mL; Sigma-Aldrich; Merck Millipore) for 30 min at 37 °C in a 24-well plate. After treatment, mitomycin C-treated stimulator cells were washed three times with RPMI (+) and centrifuged at 300× *g* for 5 min at RT. Subsequently, these cells were resuspended in DMEM (+) or the different prASC secretomes and placed into a 24-well plate (n = 10 for h-prASC secretomes and n = 7 for r-prASC secretomes). Responder cells (0.5 × 10^6^ cells; also resuspended in DMEM (+) or the different prASC secretomes) were added to each well containing stimulator cells. Together, stimulator and responder cells were cultured for seven days at 37 °C and 5% CO_2_. During this period, no medium change was performed.

In parallel, resting splenocytes or PBMCs (from the same donor as the stimulator cells; 10^6^ cells) were washed twice with RPMI (+), centrifuged at 300× *g* for 5 min at RT, resuspended in RPMI (+), and seeded onto a 6-well plate. Resting splenocytes were also incubated for seven days at 37 °C and 5% CO_2_, with no medium changes in this period. Upon completion of the 7-day incubation, the supernatants resulting from the one-way-MLR (MLR supernatants containing alloantibodies resulting from the exposure or not of the different secretomes) were collected (450 uL). Additionally, the number and viability of the resting cells were determined (Countess 3 Cell Counter (Thermo Fisher Scientific)).

The second part of the assay evaluated humoral alloimmunity using the antibody-mediated CDC assay. For it, we cultured the previously counted resting cells in the different MLR supernatants. Briefly, resting cells (0.5 × 10^5^) were resuspended in the respective MLR supernatants (50 uL) and seeded onto a 96-well plate. STD-M (+) served as a control. Resting splenocytes were cultured with the supernatants for 30 min at RT. After this, low-toxicity rabbit complement (Sanbio B.V., Cedarlane, Uden, The Netherlands) was added to each well and incubated for 2 h at 37 °C and 5% CO_2_.

To evaluate the resting splenocytes’ survival upon exposure to the different MLR supernatants (with or without the presence of secretomes) or controls (STD-M (+)), a WST-1 assay (Sigma-Aldrich) was performed, following the manufacturer’s instructions. The capacity of each secretome to mitigate alloimmunity was defined as the percentage of human PBMCs or rat splenocytes that survived upon exposure to the MLR supernatants. Cell survival was normalized to the control (STD-M (+)), set at 100.

### 2.14. Statistical Analysis

Data are expressed as means ± standard deviations (SDs). Statistical analysis was carried out in GraphPad Prism (version 9.2.0; GraphPad Software, Inc., La Jolla). For all in vitro functional assays, the effects of the different conditions, co-culturing, and the interaction between these two were analyzed by a two-way ANOVA (TWA). For this, the normality of the data was first tested using the Kolmogorov–Smirnov test. If the data were not normally distributed, the data were log-transformed before performing the TWA. Šídák’s multiple comparison test was used for post-testing. To obtain insights into the effect of the secretome from islets cultured alone, the islet secretomes from all conditions were compared to the islet normoxia-derived secretome. To obtain insights into the effect of the secretome from co-cultured islets, the co-cultured islet normoxia-derived secretome was also used for comparison. Moreover, to compare the effects of the different co-cultured islet secretomes, we compared all conditions to each other. Data were considered significantly different if *p* < 0.05.

## 3. Results

### 3.1. The Pancreatic Islet Secretomes After Different Culturing Conditions

The secretomes of in vitro-cultured human and rat pancreatic islets were characterized to assess alterations in their secretion profiles when encountering diverse stress conditions that mimic the situation during isolation and after PIT. Leveraging secretomic data, which included a comprehensive list of identified proteins for each condition, Metascape was used to discern the top 100 most-enriched pathways within each condition. All pathway enrichment analyses were conducted using the complete list of identified proteins from each condition’s secretome. Below, the five most-enriched pathways are discussed for each secretome. Furthermore, additional pathways of interest are highlighted, specifically those associated with angiogenesis, ECM, and immune responses, since these pathways are crucial for functional islet survival and PIT success. The baseline secretion signature was established using the normoxia-derived secretome for each species.

#### 3.1.1. The Human Islet Secretome

##### Proteomics of Human Normoxia-Derived Secretome

**Figure 2:**
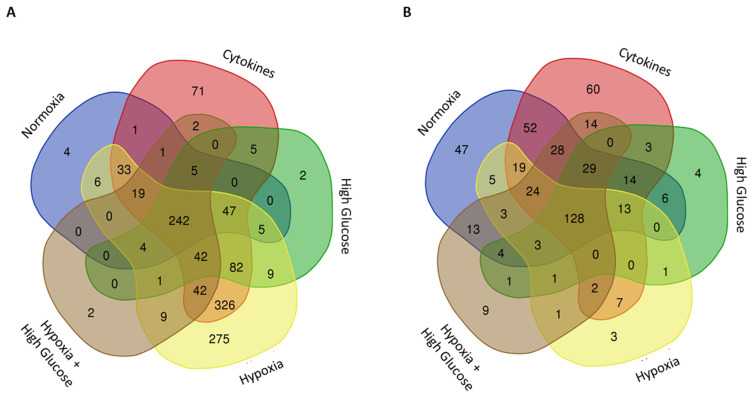
The number of human and rat proteins in islet secretomes. Global overview of human (**A**) and rat (**B**) pancreatic islet secretomes resulting from various in vitro culturing conditions.

In the normoxia-derived secretome of human islets, 367 proteins were identified (Figure 2A). The pathway enrichment analysis specific to this secretome revealed the following top five enriched pathways associated with these proteins: regulation of body fluids, wound healing, hemostasis, cellular response to toxic substances, and regulation of proteolysis (Table 2; Supplementary File S1, Table S3). Additional pathways of interest in this secretome, which are still within the top 100, encompassed cellular response to interleukin (IL)-7, chronic inflammatory response, the collagen metabolic process, regulation of humoral immune response, and blood vessel development (Supplementary File S1, Table S3).

##### Proteomics of Human Cytokine-Derived Secretome

Exposure of human islets to cytokines, mimicking an inflammatory environment, resulted in the identification of 918 proteins (Figure 2A). Among these, 570 (62.1%) were newly identified (when compared to the normoxia-derived secretome). The cytokine-derived secretome was enriched in the following pathways (top five): generation of precursor metabolites and energy, protein maturation, response to wounding, carbohydrate metabolic processes, and monocarboxylic acid metabolic processes (Table 2; Supplementary File S1, Table S4). Additionally, pathways of interest in this secretome encompassed innate immune response, cellular response to cytokine stimuli, and blood vessel development (Supplementary File S1, Table S4).

##### Proteomics of Human High-Glucose-Derived Secretome

Exposure to high glucose, simulating a hyperglycemic environment, identified 444 proteins in the islet secretome (Figure 2A). Of these, 140 (31.5%) were newly secreted compared to the normoxia-derived secretome. This secretome was enriched in pathways regulating body fluid levels, hemostasis, wound healing, generation of precursor metabolites and energy, and protein maturation (Table 2; Supplementary File S1, Table S5). Other enriched pathways included supramolecular fiber organization, innate immune response, ECM organization, regulation of complement activation, endothelial cell development, angiogenesis, and more (Supplementary File S1, Table S5).

##### Proteomics of Human Hypoxia-Derived Secretome

The exposure of human islets to hypoxia, simulating the lack of vascularization post-islet transplantation, resulted in a secretome with 1142 proteins (Figure 2A), in which 786 (68.8%) were newly secreted when compared to the normoxia-derived secretome. The human islet hypoxia-derived secretome was enriched in pathways such as generation of precursor metabolites and energy, the peptide metabolic process, cellular respiration, protein maturation, and the amide biosynthetic process (Table 2; Supplementary File S1, Table S6). Also present in this secretome were pathways of supramolecular fiber organization, ECM organization, the innate and humoral immune system, and blood vessel development, among others (Supplementary File S1, Table S6).

##### Proteomics of Human Hypoxia + High Glucose-Derived Secretome

Exposing islets to a combination of hypoxia and high glucose not only replicated physiological conditions in individuals with diabetes (hyperglycemia and insufficient oxygen supply) but also simulated a post-transplantation environment in an islet transplantation context. In the islet secretome under hypoxia and high-glucose conditions, 369 proteins were identified, and 98 of those were newly secreted when compared to the normoxia-derived secretome (26.5%) (Figure 2A). Enriched pathways included regulation of body fluid levels, wound healing, hemostasis, regulation of proteolysis, and protein maturation (Table 2; Supplementary File S1, Table S7). Other pathways of interest also identified in this secretome included supramolecular fiber organization, humoral immune response, ECM organization, blood vessel development, endothelial cell migration, and others (Supplementary File S1, Table S7).

#### 3.1.2. The Rat Islet Secretome

##### Proteomics of Rat Normoxia-Derived Secretome

In the rat islet normoxia-derived secretome, 388 proteins were identified (Figure 2B). The pathway enrichment analysis indicated their primary association with processes related to response to xenobiotic stimuli, response to metal ions, protein maturation, response to wounding, and the protein catabolic process (Table 3; Supplementary File S2, Table S3). Other pathways of interest in this secretome include response to IL-1 and IL-7, regulation of the oxygen species metabolic process, and blood vessel development (Supplementary File S2, Table S3).

##### Proteomics of Rat Cytokine-Derived Secretome

When exposed to cytokines, the rat islet secretome contained 393 proteins (Figure 2B). From those, 86 (21.9%) new proteins emerged compared to the normoxia-derived secretome. The presence of these proteins enriched this secretome in pathways of response to xenobiotic stimuli, response to metal ions, the protein catabolic process, generation of precursor metabolites and energy, and the monosaccharide metabolic process (Table 3; Supplementary File S2, Table S4). Other pathways of interest enriched in the rat islet cytokine-derived secretome includes collagen fibril organization, supramolecular fiber organization, the insulin metabolic process, cellular response to IL-1 and IL-7, and complement activation (Supplementary File S2, Table S4).

##### Proteomics of Rat High-Glucose-Derived Secretome

Exposing rat islets to high glucose yielded a secretome containing 207 proteins (Figure 2B). Only 10 (4.83%) new proteins were identified as newly secreted compared to the normoxia-derived secretome. Pathways such as protein maturation, protein folding, hemostasis, response to metal ions, and wound healing comprised this secretome’s top five most-enriched processes (Table 3; Supplementary File S2, Table S5). Other enriched pathways of interest included ECM organization, the insulin metabolic process, supramolecular fiber organization, vasculature development, the collagen metabolic process, and complement activation (Supplementary File S2, Table S5).

##### Proteomics of Rat Hypoxia-Derived Secretome

The secretome derived from rat islets exposed to hypoxia resulted in 220 proteins (Figure 2B). From those, only 15 (6.82%) new proteins were identified when compared to the normoxia-derived secretome. The top five pathways enriched included protein maturation, hemostasis, response to wounding, regulation of body fluid levels, and the hydrogen peroxide metabolic process (Table 3; Supplementary File S2, Table S6). Other pathways of interest identified in the rat islet hypoxia-derived secretome included supramolecular fiber organization and cellular response to IL-7, among others (Supplementary File S2, Table S6).

##### Proteomics of Rat Hypoxia + High Glucose-Derived Secretome

Finally, in the rat islet hypoxia + high glucose-derived secretome, 260 proteins were identified, and 28 (10.8%) of those were newly secreted when compared to the normoxia-derived secretome (Figure 2B). Pathway enrichment revealed protein folding, protein maturation, response to metal ions, the monosaccharide biosynthetic process, and response to xenobiotic stimuli as the top five highly enriched pathways in this secretome (Table 3; Supplementary File S2, Table S7). Other pathways of interest included cellular response to IL-7, collagen fibril organization, complement activation, regulation of cellular response to stress, and positive regulation of angiogenesis, among others (Supplementary File S2, Table S7).

### 3.2. The Impact of ASC and Islet Co-Culturing on the Secretome Under Different Culturing Conditions

ASCs have been shown to improve pancreatic islet function and transplantation after isolation and PIT [6,8,35]. To investigate if changes in the secretome profile might be involved in this beneficial effect, the secretomes of human and rat islets co-cultured with ASCs under various stress conditions were investigated. The secretomes of two different ratios of islets to ASCs (1:300 and 1:1000) were collected from human (Supplementary File S1, Table S8) and rat (Supplementary File S2, Table S8) co-cultures.

**Figure 3:**
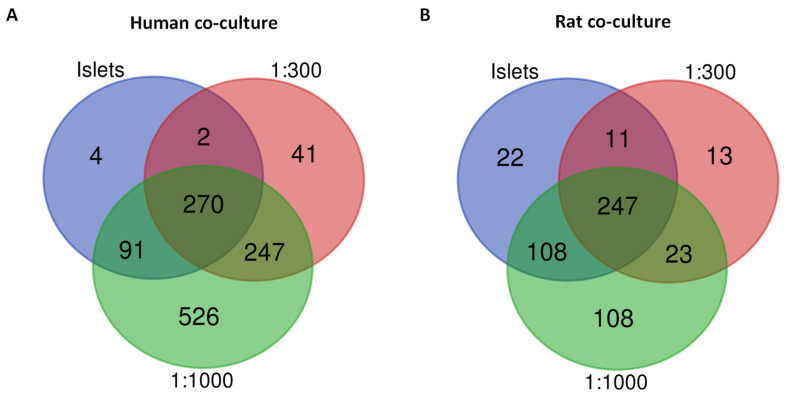
Modulation of the human and rat islet secretome compositions upon co-culturing with ASCs under normoxic conditions. Global overview of the number of proteins within the secretomes derived from human (**A**) and rat (**B**) islets co-cultured or not with adipose-derived stromal cells (ASCs; either in a 1:300 or a 1:1000 islet-to-ASC ratio).

Using the identified proteins for each condition, a pathway enrichment analysis was conducted, focusing on GO biological processes to understand the functional profile of these secretomes. Before delving into the effects of different stress conditions, we assessed the influence of the ASC proportion (1:300 or 1:1000) on the co-culture secretome composition under normoxia through a comparative analysis of total proteins in the islet-only secretome versus the islet–ASC co-culture secretome (Figure 3). This served as an indicator to discern whether a lower islet-to-ASC ratio could exert a significant influence on secretome composition.

For human islets, the 1:300 co-culture normoxia-derived secretome displayed 288 new proteins when compared to the normoxia-derived human islet secretome (constituting 78.5% of the total identified in this secretome; Figure 3A), while the 1:1000 co-culture secretome exhibited 773 newly identified proteins (68.17% of the total identified in this secretome; Figure 3A). For rat islets, the 1:300 co-culture secretome revealed 36 new proteins when compared to the rat islet normoxia-derived secretome (12.2% of the total identified; Figure 3B). In comparison, the 1:1000 co-culture secretome displayed 131 proteins (27% of the total identified; Figure 3B). Notably, a higher abundance of newly identified proteins was observed in the 1:1000 co-culture secretome compared to the 1:300 one, indicating that more pronounced changes could be identified in the functional profile of this co-culture. Consequently, our analyses focused on exploring the pathway enrichment of secretomes of 1:1000 co-cultures under both standard and stress conditions of both species.

#### 3.2.1. The Human Islet and ASC Co-Culture Secretome

##### Proteomics of Human Normoxia-Derived Co-Culture Secretome

**Figure 4:**
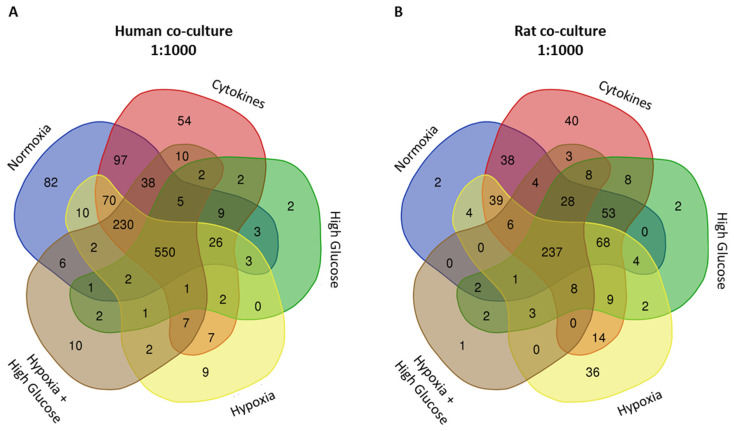
The human and rat co-cultured pancreatic islet secretomes. Global overview of the number of proteins within the secretomes derived from human (**A**) and rat (**B**) islets co-cultured with adipose-derived stromal cells in a ratio of 1:1000 under various in vitro culture conditions.

In the human co-culture normoxia-derived secretome, 1134 proteins were identified (Figure 4A; Supplementary File S1, Table S9). Comparing this secretome to the one from islets alone exposed to normoxia, 773 new proteins were found, constituting 68.1% of the total proteins identified (Figure 4A). Enriched pathways were the generation of precursor metabolites and energy, protein maturation, response to wounding, ECM organization, and regulation of proteolysis, among others (Table 4; Supplementary File S1, Table S9). Additionally, other pathways of interest were identified within this secretome. Those included the top five enriched pathways previously described in the normoxia-derived secretome from islets alone (Table 2), as well as pathways related to innate immune responses, humoral immune responses mediated by circulating immunoglobulins, complement activation, and collagen fibril organization (Supplementary File S1, Table S9).

##### Proteomics of Human Cytokine-Derived Co-Culture Secretome

In the human co-culture secretome derived under cytokine exposure, 1110 proteins were detected (Figure 4A; Supplementary File S1, Table S10). Among them, 246 new proteins were found when compared to the normoxia-derived co-culture secretome, constituting 22.2% of the total (Figure 4A). This secretome demonstrated enrichment in pathways related to protein maturation, regulation of proteolysis, the peptide metabolic process, response to wounding, and ECM organization (Table 4; Supplementary File S1, Table S10). Other pathways of interest included the top five enriched pathways previously described in the cytokine-derived secretome from islets alone (Table 2) and pathways related to innate and humoral immune responses, collagen fibril organization, and immunoglobulin-mediated immune responses (Supplementary File S1, Table S10).

##### Proteomics of Human High-Glucose-Derived Co-Culture Secretome

Under high-glucose human co-culture conditions, 611 proteins were found (Figure 4A; Supplementary File S1, Table S11), with 246 new proteins when compared to the normoxia-derived co-culture secretome, making up 40.3% of the total (Figure 4A). This secretome exhibited enrichment in pathways related to response to wounding, regulation of body fluid levels, ECM organization, extracellular structure organization, and negative regulation of peptidase activity (Table 4; Supplementary File S1, Table S11). Furthermore, additional pathways of interest included the top five enriched pathways observed in the high-glucose-derived secretome from islets alone (Table 2), as well as pathways of supramolecular fiber organization, innate immune responses, collagen metabolic processes, developments in the vasculature, blood vessel formation, and morphogenesis of blood vessels (Supplementary File S1, Table S11).

##### Proteomics of Human Hypoxia-Derived Co-Culture Secretome

In hypoxic human co-cultures, we detected 922 proteins (Figure 4A; Supplementary File S1, Table S12), and 153 (16.6%) of them were newly secreted when compared to the normoxia-derived co-culture secretome (Figure 4A). This secretome displayed enrichment in pathways linked to the response to wounding, the organization of the ECM and its structural components, protein maturation, and the regulation of body fluid levels (Table 4; Supplementary File S1, Table S12). Furthermore, additional pathways of interest included those that corresponded to the top five enriched pathways previously observed in the hypoxia-derived secretome from islets alone (Table 2), as well as humoral immune responses, the organization of collagen fibrils, developments in blood vessel formation, and humoral immune responses mediated by circulating immunoglobulins (Supplementary File S1, Table S12).

##### Proteomics of Human Hypoxia + High Glucose-Derived Co-Culture Secretome

Finally, under both hypoxia and high-glucose conditions, 869 human proteins were identified (Figure 4A; Supplementary File S1, Table S13) and 544 (62.6%) proteins were newly secreted when compared to the normoxia-derived co-culture secretome (Figure 4A). This secretome showed enrichment in pathways associated with the response to wounding, protein maturation, the regulation of body fluid levels, extracellular matrix organization, and the regulation of proteolysis (Table 5; Supplementary File S1, Table S13). Furthermore, additional enriched pathways of interest included the top five previously observed in the hypoxia + high glucose-derived secretome from islets alone (Table 2), supramolecular fiber organization, and innate immune responses (Supplementary File S1, Table S13).

#### 3.2.2. The Rat Islet and ASC Co-Culture Secretome

##### Proteomics of Rat Normoxia-Derived Co-Culture Secretome

When cultured in standard normoxic conditions, the co-culture of rat islets and rat ASCs resulted in a secretome containing 486 proteins (Figure 4B). Comparing this secretome to the one from islets alone exposed to normoxia, 131 new proteins were found, constituting 26.9% of the total proteins identified (Figure 4B). The pathway analysis unveiled significant enrichment processes, such as response to xenobiotic stimuli, response to wounding, protein folding, protein maturation, and response to metal ions (Table 5; Supplementary File S2, Table S9). Noteworthy connections were observed with the top five enriched pathways previously identified in the normoxia-derived secretome from islets alone (Table 3; Supplementary File S2, Table S3). Other pathways of interest, such as supramolecular fiber organization, generation of precursor metabolites and energy, and the ECM, were identified (Supplementary File S2, Table S9). Surprisingly, the top 100 pathways in this secretome did not include any pathways directly associated with the immune system or blood vessel development (Supplementary File S2, Table S9).

##### Proteomics of Rat Cytokine-Derived Co-Culture Secretome

The rat co-culture cytokine-derived secretome contained 563 proteins (Figure 4B; Supplementary File S2, Table S10). Among these, 187 proteins (33.2%) were newly secreted when compared to the normoxia-derived co-culture secretome (Supplementary File S2, Table S10). Enriched pathways in this mixture involved response to xenobiotic stimuli, response to metal ions, protein maturation, the protein catabolic process, and protein folding (Table 5; Supplementary File S2, Table S10). Other pathways of interest included those already described in the top five enriched pathways of the cytokine-derived islet secretome (Table 3; Supplementary File S2, Table S4), as well as supramolecular fiber organization (Supplementary File S2, Table S10). Interestingly, no immune system, blood vessel development, or additional directly ECM-associated pathways were identified within the top 100 in this secretome (Supplementary File S2, Table S10).

##### Proteomics of Rat High-Glucose-Derived Co-Culture Secretome

The investigation into the rat co-culture high-glucose-derived secretome revealed the identification of 435 proteins (Figure 4B; Supplementary File S2, Table S11). From those, 249 proteins (57.2% of the total) were newly secreted when compared to the normoxia-derived co-culture secretome (Supplementary File S2, Table S11). The pathway analysis unveiled pathways such as response to xenobiotic stimuli, response to wounding, protein maturation, protein folding, and response to metal ions as highly enriched (Table 5; Supplementary File S2, Table S11). Other pathways of interest described within the top 100 included not only the previously described top 5 most enriched of the rat islet-only high-glucose-derived secretome (Table 3; Supplementary File S2, Table S5) but also pathways of supramolecular fiber organization, regulation of endothelial cell migration, response to interleukin-1 and interleukin-6, and response to insulin, among others (Supplementary File S2, Table S11).

##### Proteomics of Rat Hypoxia-Derived Co-Culture Secretome

Rat co-cultures exposed to hypoxia secreted 431 proteins (Figure 4B; Supplementary File S1, Table S12). Among these, 249 (57.8%) were identified as newly secreted when compared to the normoxia-derived co-culture secretome (Supplementary File S1, Table S12). The pathway analysis revealed significant enrichment in response to xenobiotic stimuli, supramolecular fiber organization, protein maturation, ECM organization, and response to reactive oxygen species (Table 5; Supplementary File S1, Table S12). Other pathways of interest identified in this secretome included those already described as the top five most enriched in the rat islet-only hypoxia-derived secretome (Table 3; Supplementary File S2, Table S6). Additionally, positive regulation of endothelial cell migration, collagen fibril organization, and others were identified as enriched pathways (Supplementary File S1, Table S12). Notably, no pathways directly associated with immune system processes were included in this secretome’s top 100 most-enriched pathways.

##### Proteomics of Rat Hypoxia + High Glucose-Derived Co-Culture Secretome

Finally, in the secretome of rat co-cultures exposed to a combination of hypoxia and high glucose, we identified 303 proteins (Figure 4B; Supplementary File S1, Table S13). From these, 118 (40% of the total) were newly secreted when compared to the normoxia-derived co-culture secretome (Supplementary File S1, Table S13). The pathway enrichment analysis revealed significant involvement of these proteins in various processes, including response to wounding, protein maturation, response to xenobiotic stimuli, hemostasis, and protein folding (Table 5; Supplementary File S1, Table S13). Other pathways of interest were enriched, including the top five most-enriched pathways observed in the rat islet-only hypoxia + high glucose-derived secretome (Table 3; Supplementary File S2, Table S7), as well as ECM organization, regeneration, the collagen metabolic process, negative regulation of the intrinsic apoptotic signaling pathway, and positive regulation of vascular-associated smooth muscle cell proliferation (Supplementary File S1, Table S13).

### 3.3. Identification of Key Factors in the Secretomes of Islets Cultured with or Without ASCs

ELISA and Luminex techniques were applied to validate the presence of key proteins associated with important biological processes essential for promoting optimal PIT outcomes. Specifically, we focused on VEGF, PDGF, and bFGF, primarily linked to angiogenesis; Collagen I alpha 1, a major ECM component; and IL-1α and IL-10, representing pro- and anti-inflammatory factors. This validation was performed across different secretomes, comparing islets cultured alone versus those co-cultured with ASCs.

#### 3.3.1. The Concentration of Key Proteins in Secretomes from Human Islets Cultured with or Without ASCs

**Figure 5:**
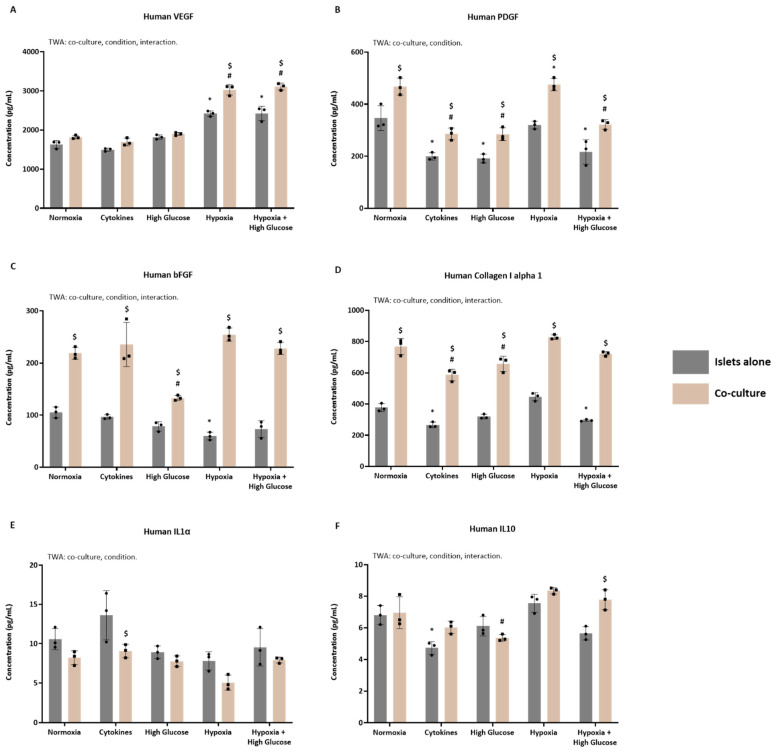
Modulation of key paracrine factors involved in pathways of interest in the human secretome upon co-culturing. Secretion of (**A**) vascular endothelial growth factor (VEGF), (**B**) platelet-derived growth factor (PDGF), (**C**) basic fibroblast growth factor (bFGF), (**D**) collagen I alpha 1, (**E**) interleukin 1 alpha (IL-1α), and (**F**) IL-10 after 72 h co-culturing under different conditions. Data represent mean values ± standard deviations of 3 pooled samples measured in triplicate. Two-way ANOVA (TWA) followed by Šídák’s multiple comparison test; * *p* < 0.05 versus normoxia islets alone; # *p* < 0.05 versus normoxia co-culture (1:1000); $ *p* < 0.05 versus respective condition islets alone.

Figure 5 illustrates the analysis of key proteins involved in the pathways of interest within the secretomes of islets cultured alone or with ASCs. For VEGF (Figure 5A), the TWA revealed significant effects of the culturing conditions (*p* < 0.0001), the presence of ASCs (*p* < 0.0001), and their interaction (*p* < 0.0001). Comparing the secretomes derived from islets cultured alone to their baseline (normoxia-derived) secretome, we observed a significant increase in VEGF in both hypoxia- (Šídák’s post-test, *p* < 0.0001) and hypoxia + high glucose-derived secretomes (Šídák’s post-test, *p* < 0.0001). Similarly, a significant increase in VEGF levels was noted when comparing the secretomes from islets co-cultured with ASCs to their baseline—both hypoxia- (Šídák’s post-test, *p* < 0.0001) and hypoxia + high glucose-derived secretomes (Šídák’s post-test, *p* < 0.0001) displayed elevated VEGF levels. Further analysis of secretomes from islets cultured with ASCs compared to those from islets cultured alone showed a significant increase in VEGF in the hypoxia-derived (Šídák’s post-test, *p* < 0.0001) and hypoxia + high glucose-derived co-culture secretomes (Šídák’s post-test, *p* < 0.0001). These results indicate that VEGF secretion is upregulated in response to hypoxia, particularly when islets are co-cultured with ASCs.

For PDGF (Figure 5B), the TWA indicated significant effects of both the culturing conditions (*p* < 0.0001) and the presence of ASCs (*p* < 0.0001). Comparing the secretomes derived from islets cultured alone to their baseline secretome, a significant decrease in PDGF levels was evident in all secretomes (Šídák’s post-test; *p* < 0.0001 for cytokines and high glucose, *p* = 0.0003 for hypoxia + high glucose) except for the hypoxia-derived secretome. Similarly, significantly decreased PDGF levels were observed in all secretomes (Šídák’s post-test; *p* < 0.0001 for cytokines, high glucose, and hypoxia + high glucose) except for the hypoxia-derived one when comparing the secretomes from islets co-cultured with ASCs to their baseline. Further analysis of secretomes from islets co-cultured with ASCs, compared to those from islets cultured alone, revealed an overall significant increase in PDGF levels in the co-culture secretomes (Šídák’s post-test; *p* = 0.0006 for normoxia, *p* = 0.0198 for cytokines, *p* = 0.0097 for high glucose, *p* < 0.0001 for hypoxia, *p* = 0.0028 for hypoxia + high glucose). Thus, while PDGF levels generally decreased under stress, co-culturing with ASCs mitigated this reduction, especially in hypoxic environments.

For bFGF (Figure 5C), the TWA demonstrated significant effects of the culturing conditions (*p* < 0.0001), the presence of ASCs (*p* < 0.0001), and their interaction (*p* < 0.0001). Comparing the secretomes derived from islets cultured alone to their baseline secretome, a significant decrease in bFGF levels was only detected in the hypoxia-derived secretome (Šídák’s post-test, *p* = 0.0393). When comparing the secretomes from islets co-cultured with ASCs to their baseline secretome, significantly decreased bFGF levels were observed only in the high-glucose-derived secretome (Šídák’s post-test, *p* < 0.0001). In the comparison of various secretomes from islets cultured with ASCs to those from islets cultured alone, an overall significant increase in bFGF levels was observed (Šídák’s post-test; *p* < 0.0001 for normoxia, cytokines, hypoxia, and hypoxia + high glucose; *p* = 0.0084 for high glucose). These findings suggest that co-culturing with ASCs promotes higher bFGF secretion, even in stressed conditions.

For collagen I alpha 1 (Figure 5D), the TWA demonstrated significant effects of the culturing conditions (*p* < 0.0001), the presence of ASCs (*p* < 0.0001), and their interaction (*p* = 0.0385). Comparing the secretomes derived from islets cultured alone to their baseline secretome, a significant decrease in collagen I alpha 1 was observed in the cytokines (Šídák’s post-test, *p* = 0.0023) and hypoxia + high glucose-derived secretomes (Šídák’s post-test, *p* = 0.0400). When comparing the secretomes from islets co-cultured with ASCs to their baseline, significantly decreased collagen I alpha 1 levels were observed in the cytokine- (Šídák’s post-test, *p* < 0.0001) and high glucose-derived secretomes (Šídák’s post-test, *p* = 0.0023). When comparing various secretomes from islets cultured with ASCs to those from islets cultured alone, an overall robust and significant increase in collagen I alpha 1 was observed (Šídák’s post-test, *p* < 0.0001 for all). These results indicate that co-culturing with ASCs enhances collagen I alpha 1 secretion, even given exposure to stressors.

For IL-1α (Figure 5E), the TWA demonstrated significant effects of both culturing conditions (*p* = 0.0004) and the presence of ASCs (*p* = 0.0002). When comparing the secretomes derived from islets cultured alone to their baseline secretome, no changes in IL-1α were identified. Similarly, no changes were found when comparing the secretomes from islets co-cultured with ASCs to their baseline. In the comparison of various secretomes from islets cultured with ASCs to those from islets cultured alone, despite a tendency towards an overall decrease, IL-1α levels were only significantly reduced in the cytokine-derived co-culture secretome (Šídák’s post-test, *p* < 0.0174). Thus, ASC co-culture slightly reduced IL-1α levels under inflammatory conditions.

For IL-10 (Figure 5F), the TWA showed significant effects of the culturing conditions (*p* < 0.0001), the presence of ASCs (*p* = 0.0018), and their interaction (*p* = 0.0028). Comparing the secretomes derived from islets cultured alone to their baseline secretome, a significant decrease in IL-10 levels was only detected in the cytokine-derived secretome (Šídák’s post-test, *p* = 0.0022). When comparing the secretomes from islets co-cultured with ASCs to their baseline, significantly decreased IL-10 levels were observed only in the high-glucose-derived secretome (Šídák’s post-test, *p* = 0.0277). In the comparison of various secretomes from islets cultured with ASCs to those from islets cultured alone, despite a tendency towards an overall increase in IL-10 levels, a significant increase was only observed in the hypoxia + high glucose-derived secretome (Šídák’s post-test, *p* = 0.0018). These findings suggest that ASC co-culture may promote IL-10 secretion under specific stressors, particularly in environments involving both hypoxia and high glucose.

#### 3.3.2. The Concentration of Key Proteins in Secretomes from Rat Islets Cultured with or Without ASCs

**Figure 6:**
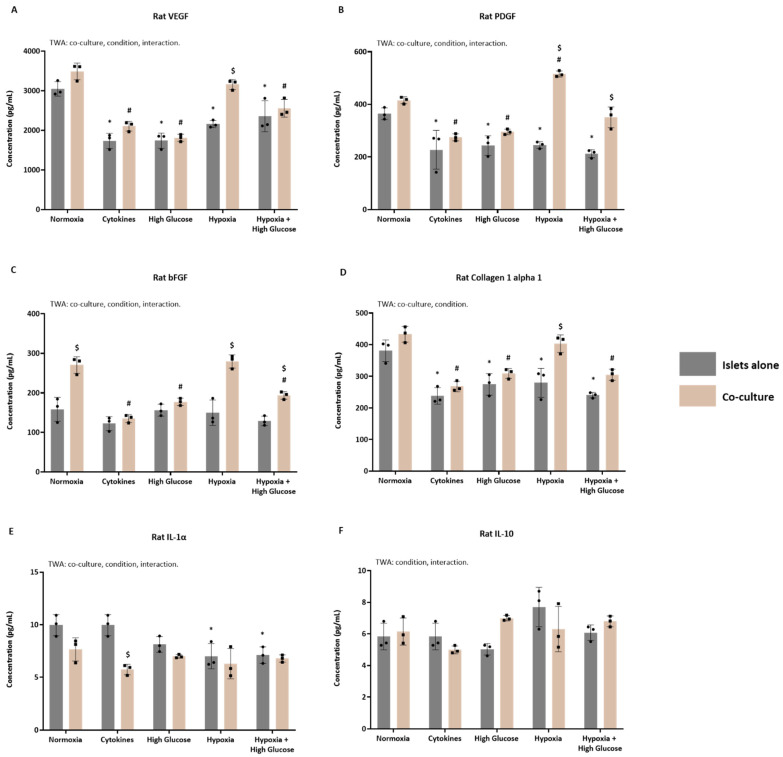
Modulation of key paracrine factors involved in pathways of interest in the rat secretome upon co-culturing. Secretion of (**A**) vascular endothelial growth factor (VEGF), (**B**) platelet-derived growth factor (PDGF), (**C**) basic fibroblast growth factor (bFGF), (**D**) collagen I alpha 1, (**E**) interleukin 1 alpha (IL-1α), and (**F**) IL-10 after 72 h co-culturing under different conditions. Data represent mean values ± standard deviations of 3 pooled samples measured in triplicate. Two-way ANOVA (TWA) followed by Šídák’s multiple comparison test; * *p* < 0.05 versus normoxia islets alone; # *p* < 0.05 versus normoxia co-culture (1:1000); $ *p* < 0.05 versus respective condition islets alone.

Figure 6 shows the analysis of key proteins involved in the pathways of interest within the secretomes of rat islets cultured alone or with ASCs. For VEGF (Figure 6A), culturing conditions (*p* < 0.0001), ASC presence (*p* < 0.0001), and their interaction (*p* = 0.0079) significantly influenced the TWA. When comparing islets cultured alone to their baseline secretome, a general decrease in VEGF levels was observed (Šídák’s post-test; *p* < 0.0001 for cytokines, *p* = 0.0004 for high glucose, *p* = 0.0056 for hypoxia, *p* = 0.0056 for hypoxia + high glucose). Islets co-cultured with ASCs exhibited a significant decrease in VEGF in all secretomes compared to their baseline (Šídák’s post-test; *p* < 0.0001 for cytokines and high glucose, *p* = 0.0002 for hypoxia + high glucose), except for the hypoxia-derived secretome. Further analysis of secretomes from islets cultured with ASCs, compared to those from islets cultured alone, revealed a significant VEGF increase only in the hypoxia-derived secretome (Šídák’s post-test, *p* < 0.0001). In summary, while VEGF levels generally decreased in the presence of ASCs, hypoxia alone induced a notable increase in VEGF in the co-cultured secretomes.

For PDGF (Figure 6B), the TWA demonstrated significant effects of the different culturing conditions (*p* < 0.0001), the presence of ASCs (*p* < 0.0001), and their interaction (*p* < 0.0001). Comparing the secretomes from rat islets cultured alone to their baseline secretome, an overall significant decrease in PDGF levels was noted (Šídák’s post-test; *p* = 0.004 for cytokines, *p* = 0.0018 for high glucose, *p* = 0.0022 for hypoxia, *p* = 0.0001 for hypoxia + high glucose). When comparing secretomes from islets co-cultured with ASCs to their baseline, again, all secretomes exhibited significantly reduced PDGF levels (Šídák’s post-test; *p* = 0.000 for cytokines, *p* = 0.0019 for high glucose, *p* = 0.0117 for hypoxia), except for the hypoxia + high glucose-derived secretome. Further analysis of secretomes from islets cultured with ASCs, compared to those from islets cultured alone, revealed a significant increase in PDGF levels in the co-culture secretomes derived from hypoxia (Šídák’s post-test, *p* < 0.0001) and hypoxia + high glucose culturing (Šídák’s post-test, *p* = 0.0004). Overall, PDGF levels generally decreased in the presence of ASCs, except for conditions involving hypoxia and hypoxia + high glucose, where significant increases were observed.

For bFGF (Figure 6C), the TWA demonstrated significant effects of the culturing conditions (*p* < 0.0001), the presence of ASCs (*p* < 0.0001), and their interaction (*p* < 0.0001). Comparing the secretomes derived from islets cultured alone to their baseline secretome, no changes in bFGF levels were identified. When comparing the secretomes from islets co-cultured with ASCs to their baseline, significantly decreased bFGF levels were observed in the cytokine- (Šídák’s post-test, *p* < 0.0001), high-glucose- (Šídák’s post-test, *p* = 0.0001), and hypoxia + high glucose-derived secretomes (Šídák’s post-test, *p* = 0.0011). In the comparison of various secretomes from islets cultured with ASCs to those from islets cultured alone, significantly increased bFGF levels were observed in the normoxia-, hypoxia- (Šídák’s post-test, *p* < 0.0001), and hypoxia + high glucose-derived secretomes (Šídák’s post-test, *p* = 0.0063). In summary, ASCs led to increased bFGF levels, especially under hypoxic conditions, although co-culture reduced bFGF under cytokine and high-glucose conditions.

For collagen I alpha 1 (Figure 6D), the TWA revealed significant effects of both culturing conditions (*p* < 0.0001) and the presence of ASCs (*p* < 0.0001). Comparing the secretomes derived from islets cultured alone to their baseline secretome, a significant decrease in collagen I alpha 1 was observed in all secretomes (Šídák’s post-test; *p* < 0.0001 for cytokines and hypoxia + high glucose, *p* = 0.0015 for high glucose, *p* = 0.0026 for hypoxia). When comparing secretomes from islets co-cultured with ASCs to their baseline (normoxia-derived secretome), significantly decreased collagen I alpha 1 levels were observed in all secretomes (Šídák’s post-test; *p* < 0.0001 for cytokines, *p* = 0.0003 for high glucose, *p* = 0.0002 for hypoxia + high glucose), except for the hypoxia-derived one. In the comparison of various secretomes from islets cultured with ASCs to those from islets cultured alone, significantly increased collagen I alpha 1 was observed only in the hypoxia-derived secretome (Šídák’s post-test, *p* = 0.0003). In summary, ASCs generally reduced collagen I alpha 1 levels, but hypoxic conditions caused an increase in collagen I alpha 1 in the co-culture secretomes.

For IL-1α (Figure 6E), the TWA indicated significant effects of the culturing conditions (*p* < 0.0001), the presence of ASCs (*p* = 0.0054), and their interaction (*p* = 0.0097). Comparing the secretomes derived from islets cultured alone to their baseline secretome, a significant decrease in IL-1α was observed in the hypoxia- (Šídák’s post-test, *p* = 0.0101) and hypoxia + high glucose-derived secretomes (Šídák’s post-test, *p* = 0.0134). In the comparison of secretomes from islets co-cultured with ASCs to their baseline, no significant changes in IL-1α levels were identified. When comparing various secretomes from islets cultured with ASCs to those from islets cultured alone, despite a tendency towards an overall decrease, IL-1α levels were only significantly reduced in the cytokine-derived co-culture secretome (Šídák’s post-test, *p* = 0.0002). In summary, ASCs did not significantly affect IL-1α levels under most conditions, except for a decrease in the cytokine-derived secretome.

For IL-10 (Figure 6F), the TWA revealed significant effects of the culturing conditions (*p* = 0.0315) and their interaction (*p* = 0.0130). When comparing the secretomes derived from islets cultured alone to their baseline secretome, no significant changes in IL-10 levels were detected. Similarly, no significant changes were identified when comparing the secretomes from islets co-cultured with ASCs to their baseline. Additionally, when comparing various secretomes from islets cultured with ASCs to those from islets cultured alone, no modulation of IL-10 was found. In summary, ASCs and different culturing conditions did not result in significant changes in IL-10 levels across the various secretomes.

### 3.4. In Vitro Functional Properties of Islet (Cultured with or Without ASC) Secretomes

Most analyzed secretomes contained proteins associated with angiogenesis, ECM composition, and immune responses. Next, we aimed to investigate in vitro the effects of the secretomes on angiogenesis, ECM composition, and immune responses using the TFA, Picrosirius Red staining, and the MLR followed by an antibody-mediated CDC.

#### 3.4.1. The Proangiogenic Potential of Human Secretomes

**Figure 7:**
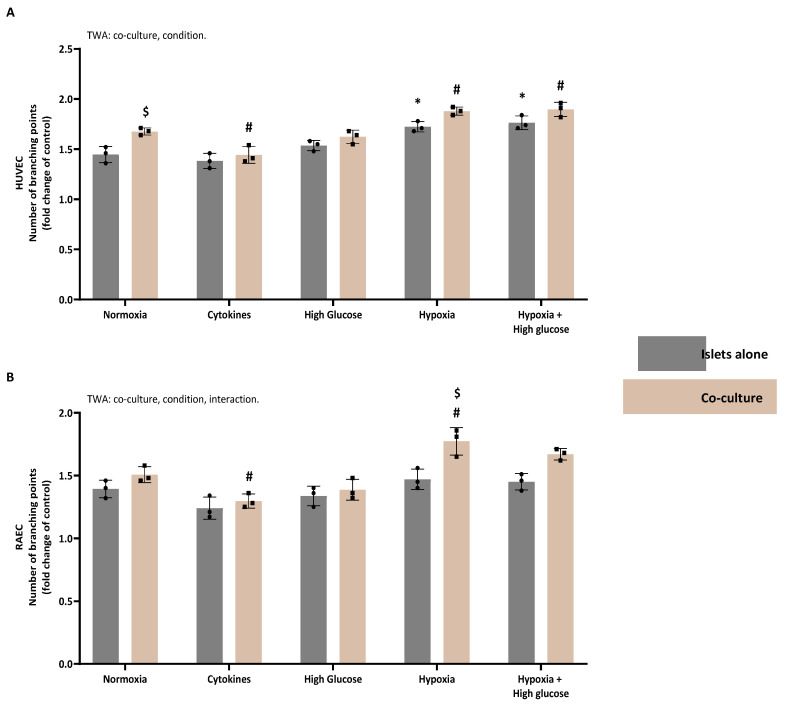
The pro-angiogenic effects of the various secretomes on human and rat endothelial cells. Quantification of the branching points formed by (**A**) human and (**B**) rat tube formation assays. HUVECs (human umbilical vein endothelial cells) or RAECs (rat aortic endothelial cells) were exposed to the various secretomes derived from human or rat islets or ASC co-culture (1:1000) for 20 h (n = 3). The number of branching points was normalized to the control (CMRL (−)), set at 1. Data are represented as individual values and means ± standard deviations. Statistical significance was assessed using two-way ANOVA (TWA) and Šídák’s multiple comparison test; * *p* < 0.05 versus normoxia islets alone; # *p* < 0.05 versus normoxia co-culture (1:1000); $ *p* < 0.05 versus respective condition islets alone.

Figure 7A illustrates the analysis of branching points resulting from the TFA with HUVECs incubated in secretomes from human islets, either alone or co-cultured with ASCs under various conditions. The TWA revealed significant effects of both culturing conditions (*p* < 0.0001) and the presence of ASCs (*p* < 0.0001). Comparing secretomes derived from islets alone under different culturing conditions to their baseline secretome, we observed a significant increase in branching points when HUVECs were exposed to hypoxia- (Šídák’s post-test: *p* = 0.0005) and hypoxia + high glucose-derived (Šídák’s post-test, *p* < 0.0001) secretomes. For co-cultured islets, the secretome derived from exposure to cytokines significantly decreased the number of branching points (Šídák’s post-test, *p* = 0.0033), while the secretomes derived from hypoxia (Šídák’s post-test, *p* = 0.0126) and hypoxia + high glucose (Šídák’s post-test, *p* = 0.0060) significantly increased branching points formed by HUVECs as compared to the normoxia-derived secretome of co-cultured islets. Notably, comparing the secretomes from all conditions, from islets cultured alone to islets cultured with ASCs, only the normoxia-derived co-culture secretome significantly enhanced the number of branching points formed by HUVECs (Šídák’s post-test, *p* = 0.0039).

#### 3.4.2. The Proangiogenic Potential of Rat Secretomes

Figure 7B depicts the analysis of branching points resulting from the TFA with RAECs, utilizing secretomes from rat islets alone or in co-culture with rat ASCs under various culturing conditions. The TWA demonstrated significant effects of the culturing conditions (*p* < 0.0001), the presence of ASCs (*p* < 0.0001), and their interaction (*p* = 0.038). When comparing different culturing conditions for islets alone, none of the secretomes affected the number of branching points differently than their baseline (normoxia-derived) secretome. In contrast, for islets cultured with ASCs, secretomes derived from cytokine exposure (Šídák’s post-test, *p* = 0.039) significantly decreased the number of branching points compared with the normoxic secretome, while hypoxia (Šídák’s post-test, *p* = 0.0048) significantly increased the number of branching points compared to the normoxic secretome. Post-testing also showed that only using hypoxia (Šídák’s post-test, *p* = 0.0012) and high glucose + hypoxia (Šídák’s post-test, *p* = 0.0271) secretomes increased the number of branching points with the secretomes from islets with ASCs compared to the secretomes of islets only.

#### 3.4.3. The Potential of Human Secretomes to Modulate Collagen Deposition

**Figure 8:**
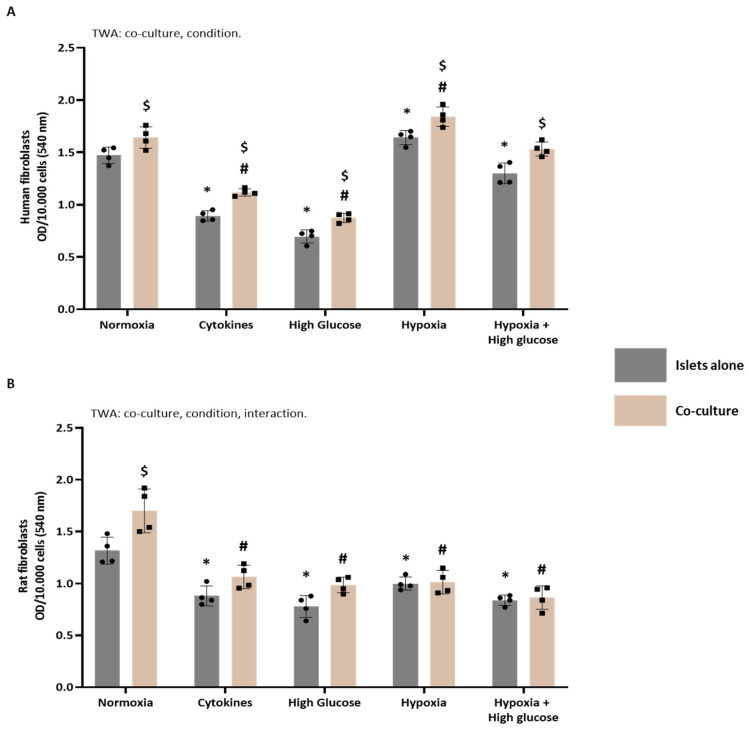
The deposition of collagen by human and rat fibroblasts exposed to the various secretomes. Spectrophotometric analysis of the Picrosirius Red staining. (**A**) Human and (**B**) rat fibroblasts exposed to various secretomes derived from human or rat islets or ASC co-culture (1:1000) for 72 h (n = 4). The data were normalized to the control (STD-M (−)), set at 1. Data are represented as individual values and means ± standard deviations. Statistical significance was assessed using two-way ANOVA (TWA) and Šídák’s multiple comparison test; * *p* < 0.05 versus normoxia islets alone; # *p* < 0.05 versus normoxia co-culture (1:1000); $ *p* < 0.05 versus respective condition islets alone.

Figure 8A presents the analysis of collagen deposition by human fibroblasts following incubation with secretomes from human islets either alone or in co-culture with human ASCs under diverse culturing conditions. The TWA indicated significant effects of both culturing conditions (*p* < 0.0001) and the presence of ASCs (*p* < 0.0001). For islets alone, compared to normoxic culturing, the cytokine secretomes (Šídák’s post-test, *p* < 0.0001), high-glucose secretomes (Šídák’s post-test, *p* < 0.0001), and high glucose + hypoxia secretomes (Šídák’s post-test, *p* < 0.0296) induced decreased collagen deposition, while the hypoxia secretome (Šídák’s post-test, *p* < 0.0327) increased collagen deposition compared to the normoxic secretome. Similarly, for islets with ASCs, compared to normoxic culturing, both cytokine secretomes (Šídák’s post-test, *p* < 0.0001) and high-glucose secretomes (Šídák’s post-test, *p* < 0.0001) induced decreased collagen deposition, while hypoxia (Šídák’s post-test, *p* < 0.0071) increased collagen deposition compared to the normoxic secretome. Post-testing also revealed that under all conditions, the secretomes of islets co-cultured with ASCs induced significantly more collagen deposition compared to islets alone (Šídák’s post-test; *p* = 0.0327 for cytokines, *p* = 0.0019 for high glucose, *p* = 0.0216 for hypoxia, *p* = 0.0013 for hypoxia + high glucose).

#### 3.4.4. The Potential of Rat Secretomes to Modulate Collagen Deposition

Figure 8B displays the analysis of collagen deposition by rat fibroblasts following incubation with secretomes from rat islets either alone or in co-culture with rat ASCs under diverse culturing conditions. The TWA indicated significant effects of both culturing conditions (*p* < 0.0001) and the presence of ASCs (*p* = 0.0001), with an observed interaction between culturing conditions and ASC presence (*p* = 0.0188). For islets alone, all secretomes significantly decreased collagen deposition compared to normoxic culturing (Šídák’s post-test; *p* = 0.0001 for cytokines, *p* < 0.0001 for high glucose, *p* = 0.0065 for hypoxia, *p* < 0.0001 for hypoxia + high glucose). Similarly, significantly decreased collagen deposition was observed for all secretomes of islets with ASCs compared with normoxia (Šídák’s post-test, *p* < 0.0001 for all conditions). Post-testing also revealed that only under normoxia did the secretome of islets co-cultured with ASCs induce significantly more collagen deposition than islets alone (Šídák’s post-test, *p* < 0.0007).

#### 3.4.5. The Immunomodulatory Potential of Human Secretomes

**Figure 9:**
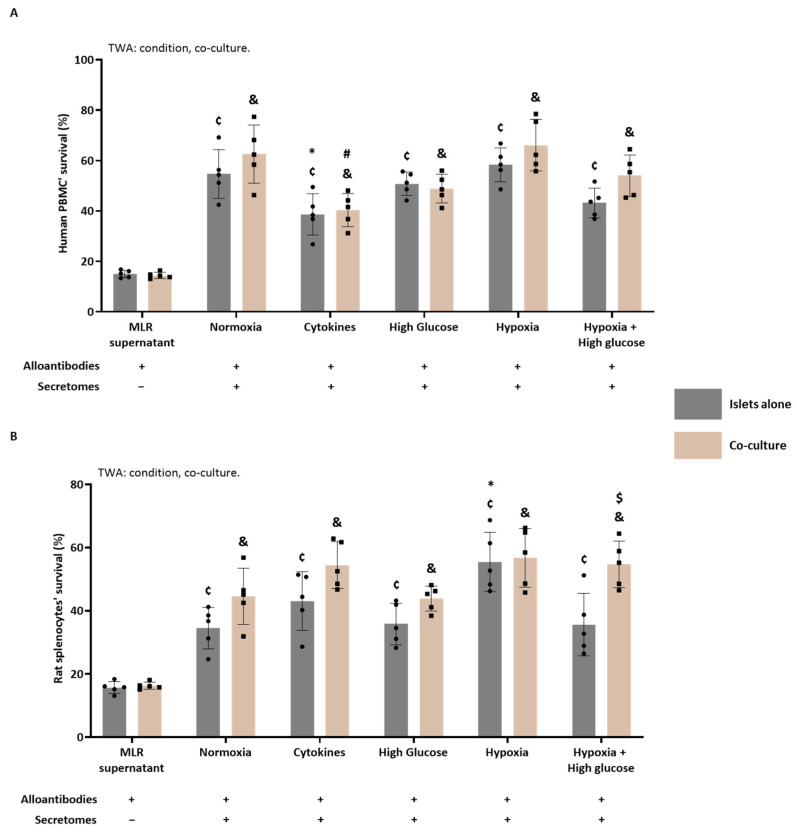
The ability of human and rat secretomes from islets and co-cultured islets to modulate antibody-mediated immune responses. The effects of various secretomes derived from (**A**) human and (**B**) rat islets and ASC co-cultures (1:1000) on humoral alloimmunity, evaluated using a mixed lymphocyte reaction (MLR) followed by an antibody-mediated cell-dependent cytotoxicity (CDC) assay. The outcome is expressed as the percentage of human peripheral blood mononuclear cells (PBMCs) or rat splenocytes that survived the exposure to the alloantibodies (n = 5 for humans and rats). CMRL (−) was used as a control, and together with the MLR supernatant it showed the efficacy of the antibody-mediated CDC assay. Cell survival was normalized to the control (CMRL (−)), set at 100. Data are represented as means ± standard deviations. Statistical significance was assessed using two-way ANOVA (TWA) and Šídák’s post hoc test; ¢ versus MLR supernatant islets alone; & versus MLR supernatant co-culture; * *p* < 0.05 versus normoxia islets alone; # *p* < 0.05 versus normoxia co-culture (1:1000); $ *p* < 0.05 versus respective condition islets alone.

Figure 9A illustrates the survival percentage of human PBMCs after the antibody-mediated CDC assay. The effectiveness of the antibody-mediated CDC assay is evident from a significant decline (*p* < 0.0001) in PBMC survival after incubation with MLR supernatant (only alloantibodies without secretomes) compared to resting PBMCs exposed solely to STD-M (+) (100% survival, data not shown in the graph).

The TWA indicated significant effects of both culturing conditions (*p* < 0.0001) and the presence of ASCs (*p* = 0.0287). MLR supernatants, generated in the presence of secretomes derived from both islets alone and co-cultured islets under various conditions, induced increased PBMC survival (Šídák’s post-test, *p* < 0.0001) compared to MLR supernatant only (containing no secretomes). The PBMC survival after incubation with the alloantibodies generated in the MLR in the presence of the different secretomes of the islets alone was compared to the PBMC survival after incubation with alloantibodies generated in the MLR in the presence of the normoxic secretome of the islets alone. This showed that the PBMC survival of the human cytokine-derived islet secretome significantly decreased compared to the PBMC survival of the normoxia-derived islet secretome (Šídák’s post-test, *p* = 0.0261). When comparing the different secretome conditions to the normoxic secretome from the co-cultured islets, the PBMC survival after incubation of alloantibodies generated in the presence of the cytokine-derived co-culture secretome was significantly decreased (Šídák’s post-test, *p* = 0.0004). No significant effect was found when comparing the islets alone with the co-culture per condition.

#### 3.4.6. The Immunomodulatory Potential of Rat Secretomes

Figure 9B shows the survival percentage of rat splenocytes after the antibody-mediated CDC assay. The efficiency of the assay was shown by the significant decline (*p* < 0.0001) in splenocyte survival after incubation with MLR supernatant (containing only alloantibodies and no secretome) when compared with splenocytes exposed solely to STD-M (+) (100%; data not shown in the graph).

The TWA indicated that there was an effect of the culturing conditions (TWA: *p* < 0.0001) and the presence of ASCs (TWA: *p* < 0.0001). MLR supernatants generated in the presence of all secretomes from both islets or co-cultured islets significantly increased splenocyte survival compared to the MLR supernatant only (containing only alloantibodies). When comparing the different secretome conditions to the normoxic secretome of the islets alone, the splenocyte survival after incubation with the hypoxia-derived islet secretome was significantly increased (Šídák’s post-test, *p* = 0.0010). When comparing the different secretome conditions to the normoxic secretome of the co-cultured islets, there was no significant difference. Post-testing also showed a significant increase in the survival of splenocytes exposed to the MLR supernatant generated in the presence of hypoxia + high glucose-derived secretomes from islets co-cultured with ASCs (Šídák’s post-test, *p* = 0.0038) compared to the ones exposed to its counterpart from islets cultured alone.

## 4. Discussion

Despite the well-documented positive effects of ASC co-transplantation with islets, such as enhanced islet viability, reduced inflammation, and improved insulin secretion [6,7,8,9,18,20,36], the exact mechanisms underlying these benefits remain unclear. In this study, we aimed to replicate the co-transplantation environment in vitro and identify the bioactive molecules secreted by islets co-cultured with ASCs, along with their associated pathways during the first 72 h post-transplantation. By focusing on early signaling events, we aimed to clarify how ASC–islet interactions support graft function and highlight potential therapeutic targets for improving co-transplantation strategies. Specifically, our findings identified key impacts on angiogenesis, ECM deposition, and immune modulation, which are critical challenges for islets post-PIT.

Under normoxic conditions, the secretome of human islets co-cultured with ASCs showed increased angiogenic capacity, stimulated fibroblast-driven collagen secretion, and improved immune modulation compared to islets cultured alone. The upregulation of key proteins like PDGF, bFGF, and collagen I alpha 1 emphasized their role in angiogenesis and ECM-related processes. Although the precise influence of ASCs on the islet secretome remains unclear due to the limitations of the experimental design, this ECM enrichment aligns with our previous findings [21], where we described a similar enrichment in ECM-associated proteins and growth factors in the secretome of ASCs cultured alone under normoxic conditions. This suggests that ECM modulation is an inherent property of the ASC secretome. ASC-driven collagen secretion may enhance the structural and metabolic environment of islets, supporting their function. In particular, increased collagen I alpha 1 secretion during co-transplantation likely plays a critical role in supporting islet survival, as previously shown in studies on encapsulated islets [37,38]. This supports the hypothesis that ASC-driven collagen secretion underpins the observed benefits of ASC co-transplantation for islet viability [7,39,40,41].

In contrast, the secretome of rat islets co-cultured with ASCs under normoxia did not show enhanced angiogenic capacity. However, it exhibited increased collagen secretion and immune response modulation compared to islets cultured alone. bFGF emerged as the primary active factor, highlighting a species-specific difference. In our earlier work [21], we identified differences between rat and human ASC secretomes under normoxia, and these distinctions were also observed in co-culture conditions. This suggests that ASC–islet interactions in rats activate alternative molecular pathways for ECM remodeling and immune modulation, achieving similar functional outcomes but through different molecular mechanisms.

When exposed to inflammatory cytokines, the secretome of human islets co-cultured with ASCs exhibited significant modulation of the ECM (evidenced by pathway enrichment and in vitro collagen deposition) and improved capacity to regulate antibody-mediated immune responses compared to islets cultured alone. PDGF, bFGF, collagen I alpha 1, and IL-1α were shown to be players involved in these functional findings. Such ECM- and immune-related enhancements are crucial, as a robust ECM and controlled immune responses are known to support islet survival and function in transplant settings [42,43,44]. These findings support the potential of ASC co-culture as a strategy to shield islets from inflammatory stress and align with our previous work, which showed that ASCs can continue to secrete essential factors like VEGF, PDGF, bFGF, and collagen I alpha 1 even when exposed to cytokine cocktails [21].

Functional assays showed no significant pro-angiogenic or ECM-stimulating effects in rat co-culture secretomes derived from cytokine exposure, even with increased VEGF secretion relative to islets cultured alone. VEGF alone may not be sufficient to trigger angiogenesis without additional factors like PDGF, which was absent in this secretome. However, immune modulation pathways were more enriched, and the ability to regulate antibody-mediated immune responses was confirmed by MLR. In addition, the pro-inflammatory cytokine IL-1α was downregulated. Our findings highlight species-specific differences in islet responses to cytokine-induced stress, which are crucial for understanding the variability in transplant outcomes. In rats, ASCs may activate NF-κB signaling [45,46], enhancing immune modulation and potentially influencing graft survival. Interestingly, pathways related to protein homeostasis—encompassing maturation, synthesis, degradation, and folding—were consistently among the top five enriched in both human and rat co-culture secretomes. This enrichment suggests a shared mechanism by which ASCs support islet survival under inflammatory stress [47,48], maintaining cellular integrity and promoting resilience during transplantation.

Under high-glucose conditions that mimicked post-transplant hyperglycemia in diabetic patients, the secretomes of human islets co-cultured with ASCs showed significant enrichment in ECM-related pathways (organization and structure), accompanied by increases in collagen I alpha 1. This enhanced collagen I alpha 1 secretion and ECM-related pathway enrichment may help counteract oxidative stress and inflammation associated with high glucose levels, thus supporting islet function [42,43,44,49,50]. By reinforcing the ECM, ASCs may create a protective niche, supporting metabolic activities and resilience against prolonged hyperglycemia, as seen in T1D patients.

In contrast, the high-glucose-derived secretome of rat islets co-cultured with ASCs showed no significant enrichment or functional activity in angiogenesis, ECM support, or immune modulation. Instead, protein maturation and folding pathways were notably enriched, suggesting an adaptive mechanism for glucose stress. Elevated glucose can induce protein misfolding, leading to cellular stress and potential apoptosis [51,52,53]. Upregulation of protein homeostasis pathways likely represents a protective adaptation to maintain islet function under hyperglycemia, offering insights into species-specific adaptations and potential targets for improving islet survival.

In hypoxic conditions, the secretome of human islets co-cultured with ASCs demonstrated significant enrichment in ECM organization and increased collagen I alpha 1 secretion. This suggests that ASCs play a crucial role in maintaining cell integrity under low-oxygen environments. Both our findings and previous studies have identified TGF-β in the hypoxia-derived ASC secretome, as well as its activation in ASC transplantation models characterized by inflammation and hypoxia [21,54,55]. This indicates that the upregulation of ECM-associated proteins in the hypoxia-derived co-culture secretome is likely mediated through TGF-β signaling. This enhanced ECM organization is critical for the survival of islets during the post-transplantation period, when hypoxia is a significant challenge [56,57,58]. Additionally, there was an observed improvement in the in vitro capacity to modulate antibody-mediated immune responses, which cannot be attributed to the immune system-related factors we investigated, IL-1α and IL-10. However, it adds support to the claim that the immunosuppressive capacity of ASCs is maintained under hypoxic conditions [12,21,59]. Remarkably, the hypoxia-derived secretome showed upregulation of proangiogenic factors, such as VEGF, PDGF, and bFGF, as seen by others [21,54,60,61], but this increase did not lead to improved HUVEC branch formation in our model—with the presence of islets—indicating that the upregulation was insufficient for functional angiogenesis. However, the rat islet co-cultures exhibited increased proangiogenic potential under hypoxia and increased VEGF, PDFG, and bFGF compared to islets alone. This enhanced angiogenic response is critical for the survival of rat islets in oxygen-deprived conditions and suggests that ASC co-culture may activate distinct pathways in rats compared to humans, thereby contributing to tissue repair and vascularization during hypoxic stress.

When exposed to the combined stress of hypoxia and high glucose, the secretome of human islets co-cultured with ASCs showed a significant enhancement in ECM-related pathways, the ability to stimulate collagen secretion, and the capacity to modulate the immune system. Key factors such as VEGF, PDGF, bFGF, collagen I alpha 1, and IL-10 were upregulated, contributing to a secretome with a potentially anti-inflammatory profile that may offer a survival advantage in transplant settings where islets encounter hypoxic and hyperglycemic conditions. In contrast, the secretome of co-cultured rat islets under the same hypoxic and hyperglycemic conditions showed enhanced proangiogenic capacity and immunomodulatory activity. However, the specific key players responsible for these effects were not identified in the analyzed factors. The rat co-cultures lacked the ability to stimulate collagen secretion and did not exhibit significant enrichment in ECM-related pathways. This highlights important species-specific differences in response to these extreme stress conditions, warranting further investigation into the molecular and environmental factors that contribute to these observed disparities.

While this study provides valuable insights into the secretome profiles of human and rat islets co-cultured with ASCs under various stress conditions, it is important to be aware of the limitations. First, this is an exploratory analysis, and the datasets presented could be reinterpreted to yield new perspectives and hypotheses for future studies. Additionally, the secretome encompasses all proteins secreted by a cell into the extracellular space, including extracellular vesicles that may contain intracellular components, which could influence the interpretation of specific protein functions. Also, the secretomic approach by itself has limitations, including detection range constraints; for a more comprehensive and quantitative analysis of secretome components, techniques such as immune assays or targeted proteomics would be necessary. Finally, while our experimental design provides valuable insights, it is important to acknowledge that the physiological conditions in vitro may not fully replicate the complexity of the in vivo environment. Despite these limitations, our study offers a robust foundation for future research into optimizing ASC–islet co-transplantation strategies.

In conclusion, our findings demonstrate the significant impact of ASC co-culture on human and rat islet secretome profiles under transplantation-relevant conditions. Overall, this study demonstrates that the ASC–islet co-culture secretome contains markers that correlate with improved microenvironmental conditions, providing valuable insights for enhancing ASC efficacy in transplantation strategies. By defining these specific molecular interactions, our findings pave the way for optimizing ASC–islet co-transplantation approaches and offer potential targets for clinical interventions. Future studies should focus on in vivo validation and the molecular pathways involved to refine the therapeutic use of ASCs, addressing remaining translational challenges.

## Figures and Tables

**Table 1:** Characteristics of the human donors and pancreatic islets used in this study.

Donor	Age	Gender	BMI (kg/m^2^)	Islet Isolation Center	Death Cause	Purity (%)	Viability (%)
1	29	M	28	LUMC	Non-cardiac	25	>80
2	52	F	22.7	ECIT	Trauma	80	90
3	52	M	25	LUMC	Non-cardiac	60	>80
4	53	F	27	ECIT	Cerebral bleeding	75	90
5	56	F	19.4	ECIT	Cerebral bleeding	70	95
6	57	F	27	ECIT	Cerebral bleeding	60	95
7	59	M	23	ECIT	Cerebral bleeding	80	95
8	63	M	21	LUMC	Non-cardiac	60	>80
9	74	F	26	LUMC	Non-cardiac	75	>80

Abbreviations: male (M), female (F), body mass index (BMI), Leiden University Medical Center (LUMC), European Consortium for Islet Transplantation (ECIT).

**Table 2:** Human islet secretomes.

	Normoxia-Derived Secretome.		Cytokine-Derived Secretome		High-Glucose-Derived Secretome		Hypoxia-Derived Secretome		Hypoxia + High Glucose-Derived Secretome	
	Pathway	LogP	Pathway	LogP	Pathway	LogP	Pathway	LogP	Pathway	LogP
Most enriched	Regulation of body fluid levels	−30.86	Generation of precursor metabolites and energy	−50.24	Regulation of body fluid levels	−33.69	Generation of precursor metabolites and energy	−57.54	Regulation of body fluid levels	−26.39
	Wound healing	−29.20	Protein maturation	−33.89	Hemostasis	−32.16	Peptide metabolic process	−40.29	Wound healing	−25.79
	Hemostasis	−27.30	Response to wounding	−33.25	Wound healing	−31.24	Cellular respiration	−38.34	Hemostasis	−21.02
	Cellular response to toxic substances	−22.05	Carbohydrate metabolic process	−31.19	Generation of precursor metabolites and energy	−22.49	Protein maturation	−35.86	Regulation of proteolysis	−20.57
	Regulation of proteolysis	−20.62	Monocarboxylic acid metabolic process	−29.97	Protein maturation	−21.25	Amide biosynthetic process	−35.83	Protein maturation	−19.97

**Table 3:** Rat islet secretome.

	Normoxia-Derived Secretome		Cytokine-Derived Secretome		High-Glucose-Derived Secretome		Hypoxia-Derived Secretome		Hypoxia + High Glucose-Derived Secretome	
	Pathway	LogP	Pathway	LogP	Pathway	LogP	Pathway	LogP	Pathway	LogP
Most enriched	Response to xenobiotic stimuli	−16.24	Response to xenobiotic stimuli	−16.81	Protein maturation	−14.24	Protein maturation	−12.43	Protein folding	−12.58
	Response to metal ions	−13.90	Response to metal ions	−13.13	Protein folding	−11.33	Hemostasis	−12.26	Protein maturation	−11.53
	Protein maturation	−12.96	Protein catabolic process	−11.99	Hemostasis	−11.14	Response to wounding	−11.58	Response to metal ions	−10.34
	Response to wounding	−12.25	Generation of precursor metabolites and energy	−11.53	Response to metal ions	−10.66	Regulation of body fluid levels	−11.50	Monosaccharide biosynthetic process	−9.76
	Protein catabolic process	−12.08	Monosaccharide metabolic process	−10.97	Wound healing	−10.23	Hydrogen peroxide metabolic process	−11.31	Response to xenobiotic stimuli	−8.78

**Table 4:** Human co-culture secretome (1:1000).

	Normoxia-Derived Secretome		Cytokine-Derived Secretome		High-Glucose-Derived Secretome		Hypoxia-Derived Secretome		Hypoxia + High Glucose-Derived Secretome	
	Pathway	LogP	Pathway	LogP	Pathway	LogP	Pathway	LogP	Pathway	LogP
Most enriched	Generation of precursor metabolites and energy	−47.40	Protein maturation	−41.19	Response to wounding	−35.85	Response to wounding	−43.38	Response to wounding	−36.89
	Protein maturation	−38.73	Regulation of proteolysis	−40.88	Regulation of body fluid levels	−31.59	Extracellular matrix organization	−41.64	Protein maturation	−34.22
	Response to wounding	−38.05	Peptide metabolic process	−39.15	Extracellular matrix organization	−28.67	Extracellular structure organization	−41.53	Regulation of body fluid levels	−34.10
	Extracellular matrix organization	−37.75	Response to wounding	−37.05	Extracellular structure organization	−28.60	Protein maturation	−37.51	Extracellular matrix organization	−32.79
	Regulation of proteolysis	−36.19	Extracellular matrix organization	−36.39	Negative regulation of peptidase activity	−28.38	Regulation of body fluid levels	−37.21	Regulation of proteolysis	−32.63

**Table 5:** Rat co-culture secretome (1:1000).

	Normoxia-Derived Secretome		Cytokine-Derived Secretome		High-Glucose-Derived Secretome		Hypoxia-Derived Secretome		Hypoxia + High Glucose-Derived Secretome	
	Pathway	LogP	Pathway	LogP	Pathway	LogP	Pathway	LogP	Pathway	LogP
Most enriched	Response to xenobiotic stimuli	−17.75	Response to xenobiotic stimuli	−19.45	Response to xenobiotic stimuli	−18.23	Response to xenobiotic stimuli	−18.45	Response to wounding	−13.81
	Response to wounding	−13.34	Response to metal ions	−15.02	Response to wounding	−15.82	Supramolecular fiber organization	−15.48	Protein maturation	−12.27
	Protein folding	−12.75	Protein maturation	−15.00	Protein maturation	−15.08	Protein maturation	−14.51	Response to xenobiotic stimuli	−12.13
	Protein maturation	−12.67	Protein catabolic process	−14.79	Protein folding	−14.88	Extracellular matrix organization	−13.22	Hemostasis	−10.76
	Response to metal ions	−12.53	Protein folding	−13.74	Response to metal ions	−12.36	Response to reactive oxygen species	−13.22	Protein folding	−10.51

## Data Availability

The proteomic data that support the findings of this study are included in this published article and its Supplementary Material files. To access the raw data of the other functional experiments, the corresponding author can be contacted.

## Supplementary Materials

The following supporting information can be downloaded at: https://www.mdpi.com/article/10.3390/cells14040302/s1.
