# Supplementary material for: Secretome Analysis of Human and Rat Pancreatic Islets Co-Cultured with Adipose-Derived Stromal Cells Reveals a Signature with Enhanced Regenerative Capacities
Source: Cells. 2025 Feb 18;14(4):302. doi: 10.3390/cells14040302 (PMC11854805; doi:10.3390/cells14040302)
Supplement: Supplementary file 1 [file cells-14-00302-s001.zip › cells-3377172 Supplementary Figure.pdf]

Supplementary Figure S1

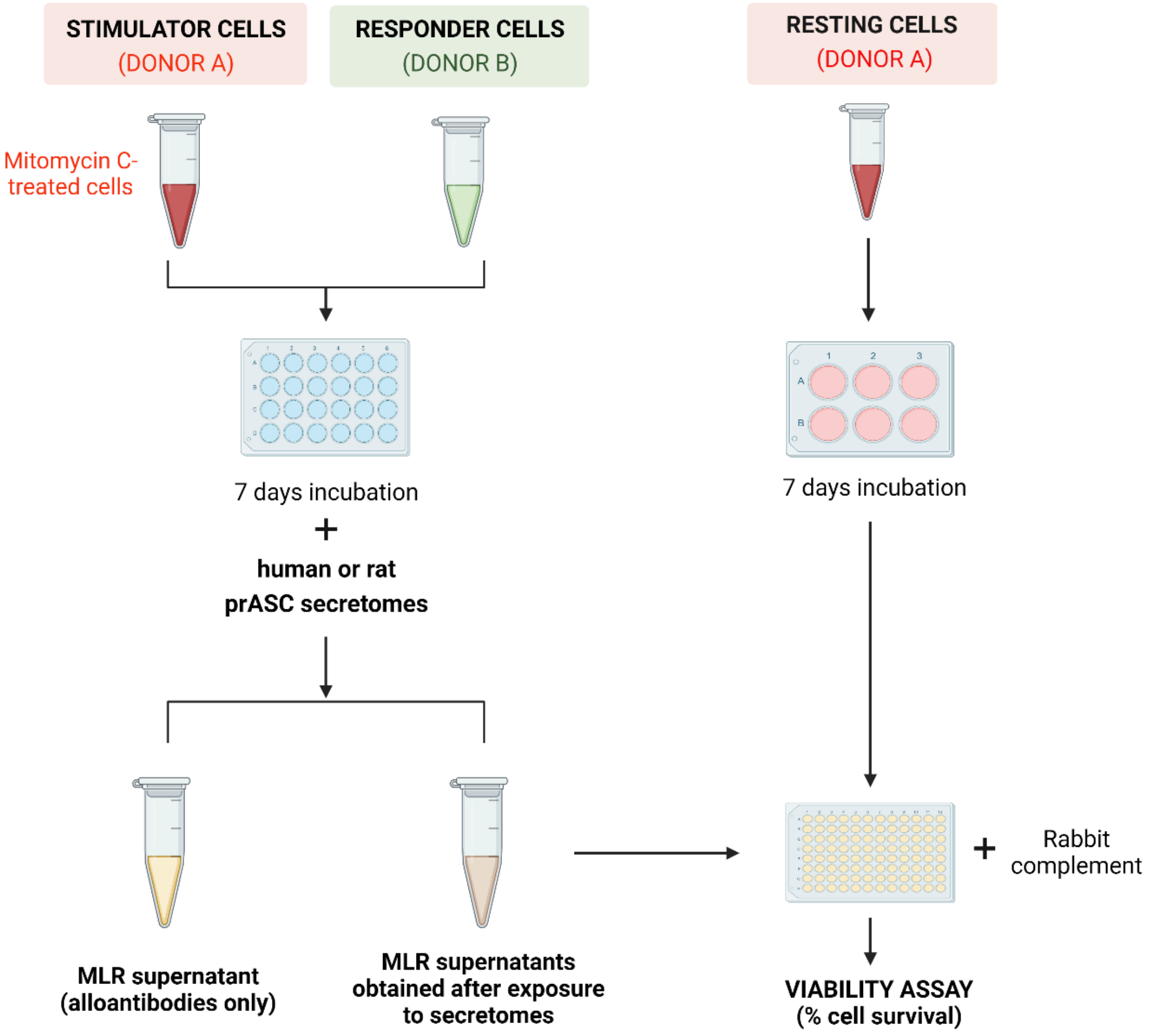

Supplementary Figure S1. Illustrative representation of the one-way mixed lymphocyte reaction (MLR) followed by an antibody-mediated cell dependent cytotoxicity assay (CDC) protocol used to evaluate the various human and rat perirenal adipose-derived stromal cells (prASC) secretomes' capacity to modulate antibody-mediate immune responses. Created with BioRender.com.
